# Environmental Pollution and Chronic Kidney Disease

**DOI:** 10.7150/ijms.51594

**Published:** 2021-01-01

**Authors:** Hui-Ju Tsai, Pei-Yu Wu, Jiun-Chi Huang, Szu-Chia Chen

**Affiliations:** 1 Department of Family Medicine, Kaohsiung Municipal Ta-Tung Hospital, Kaohsiung Medical University Hospital, Kaohsiung Medical University, Kaohsiung, Taiwan; 2 Department of Family Medicine, School of Medicine, College of Medicine, Kaohsiung Medical University, Kaohsiung, Taiwan; 3 Graduate Institute of Clinical Medicine, College of Medicine, Kaohsiung Medical University, Kaohsiung, Taiwan; 4 Research Center for Environmental Medicine, Kaohsiung Medical University, Kaohsiung, Taiwan; 5 Division of Nephrology, Department of Internal Medicine, Kaohsiung Medical University Hospital, Kaohsiung Medical University, Kaohsiung, Taiwan; 6 Department of Internal Medicine, Kaohsiung Municipal Siaogang Hospital, Kaohsiung Medical University, Kaohsiung, Taiwan; 7 Faculty of Medicine, College of Medicine, Kaohsiung Medical University, Kaohsiung, Taiwan

**Keywords:** chronic kidney disease, environmental pollution, heavy metal

## Abstract

Chronic kidney disease (CKD) is a global public health problem associated with high rates of morbidity and mortality due to end-stage renal disease and cardiovascular disease. Safe and effective medications to reverse or stabilize renal function in patients with CKD are lacking, and hence it is important to identify modifiable risk factors associated with worsening kidney function. Environmental pollutants, including metals, air pollutant, phthalate and melamine can potentially increase the risk of CKD or accelerate its progression. In this review, we discuss the epidemiological evidence for the association between environmental pollution and kidney disease, including heavy metals, air pollution and other environmental nephrotoxicants in the general population.

## Introduction

Chronic kidney disease (CKD) is a global public health issue 1-3. The reported prevalence of CKD is 11.9% in Taiwan 4, and it has gradually increased over the past decade resulting in a large economic burden on the National Health Insurance program. CKD is defined as either a reduced glomerular filtration rate (GFR < 60 mL/min/1.73 m2) or evidence of kidney damage such as an abnormal pathology or albuminuria for at least 3 months. CKD is one of the ten leading causes of death in Taiwan, and these patients have a higher risk of progression to dialysis and cardiovascular mortality. According to the US Renal Data System 5, the prevalence and incidence of CKD and end-stage renal disease (ESRD) in Taiwan are among the highest in the world.

The pathophysiology and mechanisms of worsening renal function are complex and multifactorial. In addition to the well-known risk factors for renal injury, such as aging, diabetes mellitus and hypertension, some environmental chemicals have also been shown to be important risk factors for renal injury 6-10. With the ever increasing use of synthetic compounds in all aspects of daily life, the risk to health of environmental toxins and pollutants becomes increasingly important. In particular, as the kidneys are responsible for excreting waste products from the body they are exposed to toxins and pollutants in the blood, and they are therefore susceptible to the adverse effects stemming from this exposure.

In this review, we summarize the current data regarding environmental exposure to toxins and pollutants and kidney disease. Environmental nephrotoxicants can be classified as follows: (1) metals, (2) air pollution, and (3) other non-metal exposure. We searched the PubMed (http://www.ncbi.nlm.nih.gov/pubmed) database to find published studies from January 1988 to June 2020 that investigated the relationships between environmental exposure to toxins and pollutants and CKD and/or markers of kidney injury. We performed the search using the following terms: 'environmental pollution and chronic kidney disease', 'environmental pollution and proteinuria', 'environmental pollution and albuminuria', 'environmental exposure and chronic kidney disease', 'environmental exposure and proteinuria', 'environmental exposure and albuminuria', 'air pollution and chronic kidney disease' , 'air pollution and proteinuria', 'air pollution and albuminuria, 'metals and chronic kidney disease', 'metals and proteinuria', 'metals and albuminuria'. The search was limited to research articles involving humans and those published in English. Unpublished data and abstracts were not included in this review.

## Metals

Metals are common environmental pollutants that have been associated with impaired kidney function in many epidemiological studies. Metals used in industrial processes have been shown to contaminate drinking water, food and soil, thereby increasing the risk of exposure among the general population. In the following sections, we summarize the metals that are known to have a nephrotoxic effect, including arsenic, cadmium, lead, mercury and uranium.

### Arsenic

Arsenic (As) is a highly toxic metalloid that occurs ubiquitously in the environment 11. Environmental sources of As include contaminated drinking water, pesticides, seafood, folk or alternative remedies, and products used for wood preservation 7. Acute As-induced renal intoxication has been shown to lead to acute tubular necrosis and tubulointerstitial nephritis 11, 12. In addition, chronic exposure to As has been associated with the development and progression of CKD due to As-induced oxidative stress 11, 13.

Exposure to As through the environment, occupation and diet has been reported to cause renal injury and the development of renal disease 11, 14, 15. A prospective observational study in Taiwan reported that people ingesting ≥ 50 μg/L of As in well water had a 30% increased risk of clinically recognized CKD compared to ≤ 10 μg/L 16. In addition, a community-based cross-sectional study conducted in central Taiwan reported that the risk for eGFR < 90 mL/min/1.73 m^2^ was increased by around 2-fold in people with a urine As level > 75 μg/g creatinine compared to those with a urine As level ≤ 35 μg/g creatinine 17. Moreover, Cheng et al. conducted another study in Taiwan including 8854 adults from a nationwide health screening program from 2000 to 2009 18. They found that > 50 μg/L of As in drinking water was associated with an odds ratio (OR) of 1.22 (95% confidence interval [CI]: 1.05-1.42) for the rapid progression of CKD (eGFR decline > 5 ml/min/1.73 m^2^/year). Another cross-sectional study conducted in China also found that a plasma As concentration > 0.93 μg/L was associated with eGFR < 60 mL/min/1.73 m^2^ 19. In addition, Liu et al. conducted a prospective cohort and reported that a plasma As concentration > 3.16 μg/L was significantly associated with an annual decline in eGFR among Chinese adults 20.

### Cadmium

Cadmium (Cd) is known to be nephrotoxic environmental pollutant 7, 21. Cd has a long half-life in the body, ranging from 7.4 to 16 years 11, 22. High levels of exposure can result in the accumulation of Cd in the proximal tubules of the kidney, and this has been shown to impair tubular function and protein reabsorption 23. In the general population, tobacco smoking is a main source of Cd exposure 24-26, and in non-smokers exposure commonly occurs from dietary intake of contaminated food and water 27. Occupational Cd exposure includes battery manufacturing, pigments, coatings, plastics, and copper and zinc smelting and welding 28. Urinary Cd is considered to be the most accurate measure of long-term exposure, whereas blood Cd is considered to be a measure of more recent exposure, such as exposure within the past month 8.

Clinically, Cd nephrotoxicity presents with symptoms including low molecular weight proteinuria, glucosuria, aminoaciduria, low molecular weight proteinuria, hypercalciuria and renal stones 8. Urinary Cd levels of 4-10 μg/g creatinine have been associated with increased microalbuminuria 29. Accordingly, the US Occupational Safety and Health Administration and World Health Organization (WHO) define the safe standard to be a urinary Cd concentration of < 3 μg/g and 5.24 μg/g creatinine, respectively 30. Increasing evidence has shown that chronic exposure to Cd is associated with reduced GFR and an increased risk of CKD 31. Two cross-sectional studies indicated that urinary Cd was significantly positively associated with renal tubule biomarkers including N-acetyl-β-D-glucosaminidase and β2-microglobulin in the general population in China and Korea 32, 33. In addition, blood Cd concentration has been associated with kidney function in US adults 34, 35. Madrigal et al. analyzed US National Health and Nutrition Examination Survey (NHANES) data from 2007-2012, and demonstrated that blood Cd concentration was positively associated with elevated albumin excretion in urine and inversely associated with eGFR. In their study, a stronger association was found between impaired kidney function and blood Cd concentration in the female participants compared to the male participants, and the association also differed according to hypertension 36. These findings are consistent with two studies using Korean NHANES 37, 38.

### Lead

Lead (Pb) is found throughout the environment, primarily due to human activity. Pb compounds are commonly used in gasoline, batteries, pipes, ammunition, paints, ceramic glazes, water contaminated by Pb pipes, food contaminated during processing, Pb-adulterated alcohol, and other industrial applications 11. Pb circulates in the blood, and it is either excreted by the kidneys or accumulates in bone. The half-life of Pb in the blood is around 35 days, compared to 10-30 years in bone 7.

Pb is the most common environmental nephrotoxicant, and exposure can cause oxidative stress in tubular and glomerular cells and lead to the generation of free radicals, potentially contributing to cellular apoptosis and subsequent changes in renal structure and function 39. Acute Pb toxicity (blood Pb level > 80-100 μg/dL) has been reported to cause proximal tubular injury, possibly due to cytoplasmic, mitochondrial and intranuclear inclusion bodies composed of Pb-protein complexes 40, and the clinical manifestations include glucosuria, aminoaciduria, phosphaturia, and Fanconi syndrome 41. Chronic Pb poisoning (blood Pb level > 60 μg/dL) has been reported to cause Pb nephropathy, which is characterized by glomerular sclerosis, tubular atrophy, tubulointerstitial fibrosis, and finally reduced GFR 41, 42. In addition, chronic low Pb exposure (blood Pb level < 5-10 mg/dL) has been reported to potentially contribute to the development of CKD and the progression of established CKD 43-45. The association between body Pb level and CKD has also been reported to be affected by age, sex, diabetes, hypertension, and uric acid level 46.

Two cross-sectional studies analyzed the US NHANES from 1999 to 2002 and 1999 to 2006 35, 47, and found that even low blood Pb levels may be associated with CKD. In the US NHANES 1999 to 2002, the prevalence of CKD among adults was found to be higher in those with higher blood Pb levels. The adjusted ORs of prevalent CKD increased with increasing quartiles of blood Pb level (Q1 < 1.06 mg/dL, Q2 = 1.06-1.63 mg/dL, Q3 = 1.63-2.7 mg/dL, Q4 ≥ 2.47 mg/dL) (OR = 1.49, 95% CI = 0.75-2.98; OR = 1.89, 95% CI = 1.09-3.30; and OR = 2.72, 95% CI = 1.47-5.04, respectively, for the second, third, and fourth quartiles) 47. Another analysis of the US NHANES 1999 to 2006 reported that among adults with a blood Pb level > 2.4 mg/dL, the OR for prevalent CKD was 1.56 (95% CI = 1.17-2.08) compared to adults with a blood Pb level ≤ 1.1 mg/dL 35. In addition, a NHANES from 2007 to 2012 demonstrated that a positive association between urine Pb level and an inverse association between blood Pb level and eGFR 34. Similarly, a cross-sectional study of Korean adults reported a positive association between blood Pb levels and renal dysfunction 48.

An increasing number of longitudinal studies have supported that Pb exposure contributes to an increased risk of kidney disease. Yu et al. explored the association between low-level environmental Pb exposure and renal function among 121 patients with non-diabetic CKD in Taiwan 49. After 4 years, every increase of 1 mg/dL in blood Pb level at baseline was associated with a decrease in GFR of 4.0 mL/min/1.73 m^2^ 49. In addition, a prospective population-based study conducted in Sweden investigated the association between low levels of Pb exposure and kidney function among 2567 participants who completed follow-up 50. Their results showed that the change in eGFR was higher in the participants in the third and fourth quartiles of blood Pb level (median concentrations of 29 and 46 μg/L, respectively) compared to those in the lowest quartiles 50. Moreover, the participants in the highest quartile of blood Pb level had a 49% increased risk of incident CKD compared to those in the three lower quartiles 50.

Several studies have explored the relationship between environmental Pb exposure and nephrolithiasis. Hara et al. recruited 1302 Flemish participants and reported that environmental Pb exposure was a risk factor for nephrolithiasis 51. In addition, a study of participants from five consecutive US NHANES 2-year cycles (2007-2016) showed that blood Pb level was associated with the risk of kidney stones in adults 52.

### Mercury

Exposure to mercury (Hg) compounds occurs via occupational, dietary and environmental sources, including contaminated water, fresh water fish from a contaminated source, predatory ocean fish, gold mining, smelting, burning fuel, incineration and whitening creams 7, 53. Hg exists in elemental, inorganic, and organic forms, all of which are nephrotoxic 11. Hg exposure occurs through oral, inhalation, and dermal routes, however the most common route is through the consumption of foods contaminated with Hg including seafood 8.

Hg readily accumulates in the kidneys and can contribute to both tubular and glomerular damage 54, 55. The* pars recta* of proximal tubules has been reported to be most sensitive to Hg, and it is usually the first nephron segment to be affected by exposure to Hg compounds 56. After filtration, Hg is reabsorbed by the proximal tubules, resulting in tubular toxicity presenting as low molecular weight proteinuria and enzymuria. Hg has been associated with CKD progression 44, 57, 58. In addition, clinical reports have suggested that Hg exposure can cause various renal manifestations including membranous glomerulopathy, interstitial nephritis, acute tubular necrosis and interstitial nephritis, or a minimal change in disease with nephrotic syndrome 59. In a study of adults in the US NHANES 2003-2004, Lin et al. reported a higher adjusted OR of reduced GFR (< 60 mL/min/1.73 m^2^) with increasing tertiles of blood Hg level (OR = 2.09 [95% CI = 1.11-3.96] and 2.94 [95% CI = 1.04-8.33] for tertile 2 [blood Hg level 0.66-1.64 mg/L] and tertile 3 [blood Hg level > 1.64 mg/L], respectively), compared with the lowest tertile (blood Hg < 0.66 mg/L). In addition, Nuyts et al. conducted a case-control study of occupational exposure to Hg in 272 patients with CKD and 272 controls matched for age, sex and area of residence, and found that Hg exposure was independently associated with an increased risk of CKD (OR = 5.13, 95% CI= 1.02-25.7) 60 However, a recent study of artisanal gold miners who were exposed to Hg vapor did not find an association between increased urinary Hg concentration and reduced eGFR 61. Kim et al. conducted a cross-sectional study of the Korean NHANES from 2008-2010, and reported that blood Hg levels, which reflect organic Hg exposure rather than inorganic Hg exposure, did not show a significant inverse association with eGFR after adjustments 48.

### Uranium

Environmental exposure to uranium (U) is mainly through the ingestion of contaminated groundwater, soil, and food 6. Occupational exposure also may occur through inhalation. U toxicity primarily occurs in the kidneys. Complexed U dissociates at a lower pH to release the reactive uranyl ion, which can interact with proximal tubule membranes. Urine is the primary means of quantifying exposure to U as most absorbed U is excreted in the urine within several weeks 8. The most frequently used standard for U kidney burden is the International Commission on Radiological Protection value of 3 μg/g 62.

Oral exposure to U from drinking contaminated water and occupational exposure have been associated with glucosuria, aminoaciduria, microalbuminuria, β2 microglobulinuria, phosphaturia, and hypercalciuria 63-66. Okaneku et al. demonstrated an association between urinary U (median = 0.009 ug/L) and moderate albuminuria, but no association with a decrease in kidney function 65. Wu et al. analyzed 934 hypertensive patients in China and found an inverse association between U level and eGFR in the overall population, and the association was stronger among women with high chromium exposure 67. Several studies have reported an association between exposure to U and kidney injury, but without statistical significance 68, 69.

## Air pollution

Particulate matter (PM) is a mixture of suspended liquid and solid particles in the air. It is a common air pollutant which varies widely in terms of size and chemical composition 70. PM is mostly composed of nitrates, sulfates, ammonium, other inorganic ions and metals, and may also involve biological agents such as allergens and microbial substances 71. The common health-related concerns of PM involve particles with a diameter < 10 μm (PM10) and 2.5 μm (PM2.5).

Increasing epidemiologic evidence suggests that PM is a risk factor for CKD 72. Studies in the US have reported that PM air pollution leads to a decline in GFR and is associated with the prevalence and incidence of CKD 73-76 Studies in Taiwanese and Korean adults have also observed associations between higher PM air pollution levels and reduced renal function, an increased risk of developing CKD, and the incidence of nephrotic syndrome 77-79. In addition, Mehta et al. investigated the association between longitudinal changes in eGFR and long-term exposure to PM2.5 in 669 older men. Their results showed that a 2.1 μg/m3 interquartile range higher 1-year exposure to PM2.5 was associated with a 1.87 mL/min/1.73 m2 lower eGFR and an additional annual decrease in eGFR of 0.60 mL/min/1.73 m^2^ per year 76. Moreover, Chan et al. conducted a study from 2001-2014 with 100,629 Taiwanese residents without CKD aged > 20 years, and found that every 10 μg/m^3^ increase in PM2.5 concentration was associated with a 6% increased risk of developing CKD (hazard ratio = 1.06, 95% CI = 1.02,1.10) 77.

Few studies have examined the association between PM and the progression of CKD in patients with kidney damage 74, 80. Bowe et al. demonstrated a significant association between PM2.5 concentration and the risk of developing kidney disease and progression to ESRD in an observational cohort of 2,482,737 US veterans 74. Another study from Hong Kong reported a positive association between annual exposure to PM2.5 and mortality from ischemic heart disease among older patients with CKD 80. In addition, a nationwide, multicenter, prospective cohort of Korean ESRD patients indicated that increased exposure to PM10 from 1 to 7 years increased the risk of mortality, and that long-term exposure to NO_2_ and SO_2_ was a significant risk factor for mortality due to ESRD regardless of the length of exposure 81. Moreover, experimental laboratory evidence has shown that exhaust particle exposure can lead to changes in hemodynamics in the kidneys, induce oxidative stress, inflammation, and DNA damage in renal tissues, worsen acute kidney injury, and further promote chronic renal injury in murine models 82, 83.

In summary, experimental and clinical findings indicate the biologic plausibility and support the hypothesis that environmental exposure to elevated levels of PM2.5 is associated with an increased risk of kidney disease. More evidence from a variety of regions and populations is needed to clarify the effects of PM on renal health. These findings support the global strategy to reduce air pollution and prevent the development of CKD.

## Other non-metals

### Phthalates

In 2011, a major health scandal involving phthalate-tainted foodstuffs occurred in Taiwan 84-90. Phthalates, and mainly di-(2-ethylhexyl) phthalate (DEHP) and/or di-isononyl phthalate (DINP) were intentionally added to foodstuffs as a substitute for emulsifiers, particularly in nutrient supplements and probiotics regularly taken by children 84-90. In addition, phthalates are commonly added to cosmetics as a solvent for fragrance and to many other commonly used products such as paint, toys, and medical devices to make them soft and flexible. Another main source of exposure to phthalates in the general population is from plastic containers or plastic bags exposed to high temperatures, and the use of plastic materials during food production 91, 92. Therefore, humans are potentially exposed to phthalates via inhalation, absorption through the skin, or ingesting food.

Recent studies have investigated the relationship between phthalate exposure and renal function including early renal injury markers such as urine albumin/creatinine ratio and urinary β2-microglobulin (β2M) in different populations. Trasande and colleagues investigated the relationship between exposure to phthalates and renal function in 667 children aged 6-19 years who participated in the 2009-2010 NHANES by measuring metabolites in their urine 93. They found that certain metabolites of high molecular weight phthalates such as DEHP in urine were significantly and positively associated with urine albumin to creatinine ratio (ACR), which is regarded to be a clinical marker of glomerular injury. After the scandal of phthalate-tainted foodstuffs in Taiwan, Tsai et al. reported a possible association between DEHP and an increase in microalbuminuria in children who consumed higher amounts of foods contaminated with phthalates 88. Chen et al. also reported associations between some DEHP metabolites and benzyl butyl phthalate and impaired renal function in 1663 adults in the 2012 Shanghai Food Consumption Survey 94. The same authors used principal component analysis to examine associations between the patterns of exposure and impaired renal function, and found positive associations between high molecular weight phthalate pattern score and renal function parameters (ACR, β2M, and N-acetyl b-D-glucosaminidase [NAG]), which is consistent with the results of single metabolite analyses 95. In addition, a study of healthy Korean female adults revealed a significant positive association between exposure to mono-n-butyl phthalate (MnBP) and urine ACR with a dose-response relationship 96. In addition, a cross-sectional study of Italian patients with diabetes mellitus revealed an association between exposure to DEHP metabolites including mono-(2-ethylhexyl) phthalate (MEHP) and mono-(2-ethyl-5-oxohexyl) phthalate (MEOHP) and the degree of albuminuria, however no association with eGFR was noted 97. In contrast, Malits et al. used a cross-sectional study design to compare the Chronic Kidney Disease in Children Study and NHANES 2007-2008, and found that children aged 1 to 17 years with CKD had not been exposed to increased levels of phthalates 98. In summary, several cross-sectional studies have suggested that phthalates may be associated with renal injury markers. Further longitudinal research is needed to clarify the causal relationship.

### Melamine

Melamine is a synthetic organic base used in many commercial products including dry erases boards, cleaning supplies, and other plastic goods. Despite the 2008 melamine baby formula scandal in China which resulted in kidney-related disease in children 99, melamine is still widely present in the environment and is detected in most urine samples obtained from the general populations of the USA and Taiwan 100, 101. A series of epidemiological studies support the hypothesis that long-term environmental exposure to low-dose melamine can increase the risk of adverse kidney outcomes, including urolithiasis, early renal damage, and the deterioration of kidney function in adults 102-106. Liu et al reported that chronic low-dose melamine exposure was associated with an increase in the risk of calcium urolithiasis formation in Taiwanese adults 102, 103, and urinary melamine has been significantly associated with NAG in urolithiasis patients, especially those with a first stone episode 105. In addition, Wu et al. reported a positive association between urinary melamine levels and urinary NAG levels, and that the detectable rate of β2M was increased in workers in melamine tableware manufacturing factories 104. Moreover, Tsai et al. conducted a prospective cohort study including patients with an eGFR ≥ 30 ml/min/1.73 m^2^ from 2006 to 2010 in Taiwan, and found that urinary melamine level was significantly associated with deterioration in kidney function in patients with early-stage CKD 106. Furthermore, Jianqiu et al. analyzed associations between melamine and cyanuric acid exposure and markers of kidney function in adults from the NHANES 2003-2004 107. They found that adults who had exposed to high levels of melamine had a lower eGFR than those who had been exposed to low levels of melamine, although there were no significant associations between melamine and cyanuric acid exposure and markers of kidney function 107. In addition, a cross-sectional study of 109 children (aged 4 months to 8 years) in the USA suggested that cyanuric acid, which is a structural analogue of melamine, was associated with increased kidney injury molecule-1 (KIM 1) concentrations 108.

Melamine has been shown to crystallize in distal renal tubules, and this may explain the reported cases of nephrolithiasis and acute kidney injury. It has also been hypothesized that melamine-induced oxidative stress contributes to renal tubular injury 109, 110. Further prospective cohort studies are needed to clarify the causal effect of environmental low-dose melamine exposure and adverse kidney outcomes in patients with stones and the detailed mechanisms leading to the adverse effects.

### Bisphenol A

Bisphenol A (BPA) is a synthetic chemical comprised of two phenol rings connected by a methyl bridge, to which two methyl groups are attached. Environmental BPA exposure can occur via absorption through the skin, ingestion and respiration, and detectable levels of BPA have been reported in the urine of >93% of adults 111, with high serum levels among men and smokers 112.

Previous studies have reported inconsistent associations between BPA and kidney function. Urinary BPA levels have been positively associated with ACR among US children 113 and Chinese adults 114. In addition, a positive association between urinary BPA levels and eGFR was found among a general population of US adult females, but not in adult males who participated in NHANES 2003-2006 115. In contrast, no associations between urinary BPA and eGFR or urine protein to creatinine ratio (PCR) or urine ACR were found among US children with CKD 98 and Korean healthy women 96. Positive associations between serum BPA level and CKD have been reported in several prospective studies of patients with type 2 diabetes 116, 117. Different characteristics of the studied populations among these published studies may at least partially explain the discrepancies in the findings.

## Conclusion

Environmental pollutants including heavy metals, PM, and other chemicals such as phthalates, melamine and BPA are important factors in the etiology of CKD, especially in developing countries in which environmental pollution is prevalent. The pathogenic mechanisms by which most environmental nephrotoxicants induce CKD have been elucidated. Most studies on the pathogenic mechanisms of environmental pollutants have focused on systemic inflammation and oxidative stress, and the detailed mechanisms of the pathogenesis of specific kidney diseases are still not fully understood. Studying interactions between environment pollutants and genetic factors may help to elucidate disease susceptibility.

Most of the epidemiological evidence regarding the association between environmental pollution and kidney diseases discussed in this study comes from cross-sectional studies. To establish causal relationships and dose-response associations between exposure to environment pollutants and kidney disease for a wide range of exposure levels, more detailed longitudinal studies and also experimental designs with specific and quantified measurements of environmental exposure are required. It is crucial to implement environmental protection strategies and establish safe exposure levels of environmental pollutants, such as air quality standards. In summary, our findings support the need for regulatory strategies for the control of pollution and reduction or prevention of exposure to environmental health risks. Clinicians should be aware of the adverse renal effects induced by environmental exposure to pollutants. Detailed exposure assessments based on the sources of exposure for potential nephrotoxicants should be performed on a patient-by-patients basis.
